# Complete denture hygiene solutions: antibiofilm activity and effects on physical and mechanical properties of acrylic resin

**DOI:** 10.1590/1678-7757-2020-0948

**Published:** 2021-09-03

**Authors:** Millena Mangueira ROCHA, Adrianne Moura CARVALHO, Flávia Cristina Targa COIMBRA, Carolina Noronha Ferraz de ARRUDA, Viviane de Cássia OLIVEIRA, Ana Paula MACEDO, Cláudia Helena SILVA-LOVATO, Valéria Oliveira PAGNANO, Helena de Freitas Oliveira PARANHOS

**Affiliations:** 1 Universidade de São Paulo Faculdade de Odontologia de Ribeirão Preto Departamento de Materiais Dentários e Prótese Ribeirão PretoSP Brasil Universidade de São Paulo, Faculdade de Odontologia de Ribeirão Preto, Departamento de Materiais Dentários e Prótese, Ribeirão Preto, SP, Brasil.

**Keywords:** Complete denture, Acrylic resin, Biofilm, Properties, Products with antimicrobial action

## Abstract

Appropriated denture hygiene is a predictive factor for longevity of rehabilitation treatment and maintenance of the oral mucosal health. Although, disinfectant solutions are commonly used as denture cleansers, the impact of these solutions on acrylic resin-based dentures remain unclear.

**Objective:**

To evaluate, *in vitro*, the antibiofilm activity of complete denture hygiene solutions and their effects on physical and mechanical properties of acrylic resin.

**Methodology:**

For antibiofilm activity measurement acrylic resin specimens were contaminated with *Candida albicans, Candida glabrata* and *Streptococcus mutans*. After biofilm growth, the specimens were assigned to the hygiene solutions: Distilled water (Control); 0.2% Sodium hypochlorite (SH); Efferdent Power Clean Crystals (EPC) and 6.25% *Ricinus communis* (RC). The viability of microorganisms was evaluated by agar plate counts. In parallel, physical, and mechanical properties of the acrylic resin were evaluated after simulating a 5-year period of daily immersion in the previously mentioned solutions. The changes in surface roughness, color, microhardness, flexural strength, impact strength, sorption and solubility were evaluated. Data were compared by ANOVA followed by the Tukey test or Kruskal-Wallis followed by the Dunn test depending on the distribution (α=0.05).

**Results:**

Regarding antibiofilm action, SH eliminated all microorganisms while EPC and RC exhibited moderate action against *S. mutans* (p=0.001) and *C. glabrata* (p<0.001), respectively. Relative to effects on the physical and mechanical properties of the acrylic resin, RC led to higher values of color change (p=0.030), hardness (p<0.001), surface roughness (p=0.006) and flexural strength (p<0.001). Moreover, RC induced the highest values of changes in solubility (p<0.001). EPC promoted greater changes in surface morphology, whereas immersion in SH retained the initial appearance of the acrylic resin surface. All hygiene solutions reduced the impact strength (p<0.05).

**Conclusion:**

SH presented the most effective antibiofilm activity. In addition, changes on properties were observed after immersion in RC, which were considered within acceptable limits.

## Introduction

The denture biofilm must be removed daily means of proper cleaning, since it can lead to local and systemic diseases.^1,2^ Sodium hypochlorite and alkaline peroxide solutions are widely indicated for denture biofilm control, in short or long-time immersions, either associated with mechanical methods such as brushing, or not.^3^ These solutions must be effective without being deleterious to the materials of which the prosthetic device is made. In addition, the type of denture cleanser, manufacturer’s instructions and period of use/immersion must be considered.

*In vitro* studies have shown the effectiveness of 1% and 0.5% sodium hypochlorite, used in different immersion periods, relative to their ability to remove biofilm and antimicrobial action against *Candida* spp.^4-6^*In vivo* studies have confirmed that 1%, 0.5% and 0.25% sodium hypochlorite solutions were efficacious for removing biofilm from denture surfaces, in addition to exhibiting significant antimicrobial activity against *Streptococcus mutans, Candida* spp. and gram negatives bacteria.^7-11^ However, adverse effects on acrylic resin-based dentures have been reported after applying 1% and 0.5% sodium hypochlorite in routine hygiene practice.^12-19^ Randomized clinical studies have shown the effectiveness of 0.2% sodium hypochlorite, with reduction in biofilm levels, notable antimicrobial action against *Candida* spp. and remission of denture stomatitis, without significant changes in color, surface roughness, and flexural strength of the acrylic resin-based dentures.^20-22^ Therefore, this solution should be evaluated against different microorganisms of which the denture biofilm is composed, such as *Candida* spp. and *S. mutans*, together with its effect on other relevant acrylic resin properties.

Alkaline peroxide solutions have been shown to be effective in short^22-30^ and long^19,31^ periods of immersion. The antimicrobial action of these solutions has been evaluated against monospecies biofilms, composed mainly of *C. albicans*.^4,32,33^ As regard adverse effects, changes in color, surface roughness and flexural strength of acrylic resin have been reported;^14,16,34^ therefore, it is important to follow the manufacturer’s instructions for better action and prevention of significant effects on prosthetic devices. The effectiveness of Efferdent peroxide-based solution against microorganisms related to local and systemic diseases has been established;^27,29^ however, the effects on the acrylic resin-based dentures properties have not yet been evaluated. Therefore, new investigations should be conducted, in order to clarify whether Efferdent peroxide-based solution could promote adverse effects on prosthetic devices.

Although *Ricinus communis* solutions have been used as denture cleansers,^6,7,10,20,35^ scientific data in the literature are controversial, and up to now, no ideal concentration has been established. A 2% solution showed moderate ability to remove denture biofilm, and effective action in reducing *C. albicans* and *S. mutans* on the surface of a complete denture reliner.^7,35^ A 3.3% mouthwash resulted in remission of denture stomatitis; however, it was unable to reduce *Candida* spp.^36^ Solutions at 8% and 10% led to a decrease in microbial load of *C. albicans, Candida glabrata,* and *S. mutans*, and remission of denture stomatitis, with moderate action on biofilm removal.^6,10,11,20,21^ In addition, the deleterious effects of these solutions on acrylic resin-based dentures properties were classified as being clinically acceptable.^15,18,21^ A previous study showed that the minimum inhibitory concentration of *R. communis* necessary to inhibit the growth of microorganisms such as *C. albicans* and *C. glabrata* was 6.25%,^22^ but studies about its safety relative to effects on resin properties have not yet been conducted.

The literature has shown the importance of using chemical cleansers in denture hygiene and the feasibility of using diluted hypochlorite,^6-11,20-22^ peroxide-based solutions^19,22,24,26,28,32^and *R. communis* solutions^6,7,10,20,22,35^in patients’ daily hygiene. Thus, studies involving these solutions should be developed, to indicate a safe protocol for patients’ health, which do not promote adverse effects on acrylic resin-based dentures. This, in turn, is one of the main factors that will ensure the long-term durability of oral rehabilitation. Therefore, in this study, *in vitro* analysis was performed to evaluate the antibiofilm activity of 0.2% sodium hypochlorite, Efferdent peroxide-based solution and 6.25% *R. communis* solution against monospecies biofilms composed of *C. albicans, C. glabrata* and *S. mutans*. In parallel, the effects on physical and mechanical properties of thermally activated acrylic resin were evaluated by simulating an estimated period of use of a complete denture. The null hypothesis was that there would have no difference on both antimicrobial action and effects on properties of acrylic resin regarding the use of the cleanser solutions.

## Methodology

### Experimental Design

In this study, the ability of the hygiene solutions to remove *C. albicans, C. glabrata* and *S. mutans* mature biofilm, grown on acrylic resin surface, was evaluated by simulating a single short daily period of immersion. In addition, denture cleanser effects on physical and mechanical properties of acrylic resin were analyzed by simulating a 5-year period of daily immersion. For both antibiofilm activity and physical/mechanical analysis, the specimens were randomly assigned to four groups according to the following hygiene solutions: I) Distilled water (Control); II) 0.2% Sodium Hypochlorite [SH - (Inject Center Manipulation Pharmacy, Ribeirão Preto, São Paulo, Brazil)]; III) Efferdent Power Clean Crystals (EPC – Medtech Products, Irvington, New York, USA); IV) 6.25% *Ricinus communis* (RC – Institute of Cheminstry of São Carlos, São Carlos, São Paulo, Brazil). The active substances of the Efferdent Power Clean Crystals are sodium perborate and ethylenediaminetetraacetic acid (EDTA) tetrasodium, which are responsible for the release of active oxygen, promoting antimicrobial and stain removal effect.^22^ In Groups Control, SH and RC, immersions were performed at room temperature, whereas in Group EPC, immersions were performed in accordance with the manufacturer’s instructions (3 min / 37 ± 2 °C). In the 5-year immersion simulation performed for physical/mechanical analysis, one hour was considered to represent 3 immersions of 20 min, thus every 24 h corresponded to 72 immersions. Therefore, to complete the period (1825 days), 25.3 days were necessary.^17^ Considering 3-minute immersion, one hour represented 480 immersions, thus 3.8 days were required to complete the period. The specimens were evaluated before and after this immersion protocol.

### Specimen preparation

Circular (15×3 mm), rectangular (65×10×3.3 mm) and disc-shaped (50×0.5 mm) metal matrices were invested with type IV dental stone (Gesso Rio, Rio Claro, São Paulo, Brazil) in a conventional denture flask. Afterwards, heat-polymerized acrylic resin (Classico, Campo Limpo Paulista, São Paulo, Brazil) was manipulated, packed, and pressed into the mold and polymerized by immersion in water (73°C for 90 min and boiling for 30 min), in an electric thermopolymerizing device (Thermocycler T100; University of São Paulo, Ribeirão Preto, São Paulo, Brazil). The specimens were deflasked and immersed in distilled water at 50°C for 24 h in order to eliminate residual monomer. Excess acrylic resin was removed with a bur (Maxi-Cut; Malleifer Instruments, Ballaiguer, Switzerland) and a micromotor (Moto Torre; EDG, São Carlos, São Paulo, Brazil). All specimen surfaces were polished in a horizontal polisher (Aropol E; Arotec, Cotia, São Paulo, Brazil) with abrasive paper (Norton Indústria Brasileira, Guarulhos, São Paulo, Brazil).

### Antibiofilm Activity

Antibiofilm activity was evaluated in triplicate, in three independent time intervals (n=9), against three strains from the American Type Culture Collection (ATCC): *Candida albicans* (10231), *Candida glabrata*, (2001) and *Streptococcus mutans* (25175). Firstly, stock cultures of frozen yeasts and bacteria were streaked out on Sabouraud Dextrose Agar (Himedia, Mumbai, India) plates and Brain Heart Infusion Agar (Himedia) plates, respectively. After incubation at 37°C for 48 h one colony was transferred to 10 ml of broth medium and incubated at 37°C to obtain exponentially growing cells. Afterwards, the tubes were centrifuged (Eppendorf, Hamburg, Germany) and the cell pellet was washed twice in phosphate-buffered saline (PBS). Optical density of *S. mutans* suspensions was verified in a spectrophotometer (Multiskan GO; Thermo Scientific, Waltham, Massachusetts, USA) at wavelength of 625 nm. Due to the variable morphology of the genus, yeast cell density was verified using a Neubauer chamber (Precicolor; HBG Henneberg-Sander, Gießen, Germany).

Monospecies biofilms were grown according to Paranhos, et al.^29^ (2019). Briefly, the circular specimens, sterilized by microwave irradiation [127 V, 800 W, 2,450 MHz (Perfect; Panasonic, Kadoma, Japan), at 650 W for 6 minutes, were aseptically distributed into 12-well tissue culture plates (TPP Techno Plastic Products, Trasadingen, Switzerland). Each well received 2 ml of medium broth containing standardized cell suspension (10^6^ CFU/ml) of *C. albicans, C. glabrata* or *S. mutans*. The plates were incubated at 37°C, at 75 rpm for 48 h to promote biofilm maturation. After incubation, the specimens were randomly assigned, and the proposed hygiene protocols were applied concurrently on three specimens with the monospecies biofilm. Specimens was transferred to a stainless-steel basket (6 cm×3 cm×2 cm) with 6 square compartments (1.5 cm×1.5 cm) and immersed in a container with 200 ml of the respective cleansing solutions.^23^ For Group EPC, one sachet of powder was added to the sterile distilled water (37±2°C). After immersion, specimens were washed with PBS, transferred to 10 ml of Letheen broth (HiMedia) and sonicated (200W, 40KHz) (Altsonic Clean; Alt equipamentos, Ribeirão Preto, São Paulo, Brazil) for 20 min. Serial dilutions aliquots (10^1^ – 10^3^) of the resulting suspension were seeded, the number of colonies was registered, and the CFU/ml value was calculated.

After hygiene procedure, two specimens of each group were fixed with 2.5% glutaraldehyde for 60 min and subsequently dehydrated in a graded ethanol series (30%, 50%, 70%, 90% and 100%). The specimens were sputter-coated with a layer of approximately 100 nm gold and positioned in a Scanning Electron Microscope (EVO 10; CARL ZEISS, Jena, Germany). Surface morphology of the biofilms was examined at 3000× magnifications under high vacuum.

### Effect on physical and mechanical properties of acrylic resin

#### Surface roughness

The roughness of rectangular specimens was evaluated with a rugosimeter (Surftest SJ-201P; Mitutoyo, Tokyo, Japan) (n=20) and a 3D laser confocal microscope (OLS4000; Olympus Tokyo, Japan) (n=3). Using the rugosimeter, three readings were performed (4 mm in length) for each specimen, and the cut-off value was 0.8 mm at a speed of 0.5 mm/s. The roughness of each specimen was calculated by the arithmetic mean of three measurements (μm). Values within 0.20 µm were considered clinically acceptable.^17,21^ For analysis under the 3D laser confocal microscope, the specimens were placed in a parallel position and 3 random images were captured. The images were obtained with a 5× objective, at a final magnification of 108×, and the mean roughness of each image (Sa) was calculated.

#### Color Change

The color measurements (n=20) were made on circular specimens by using a colorimeter (Color-guide 45/0; BYK-Gardner, Geretsried, Germany) as previously described.^17^ The CIELAB color scale was used to calculate change in color of each specimen using the ΔE equation {ΔE*=[(ΔL*)^2^+(Δa*)^2^+(Δb*)^2^]½}. The data were also quantified according to the National Bureau of Standards (NBS) units (NBS units = ΔE × 0.92) and changes were then classified according to: 1) Trace, 0.0-0.5; 2) Slight, 0.5-1.5; 3) Noticeable, 1.5-3.0; 4) Considerable, 3.0-6.0; 5) Very, 6.0-12.0; 6) Excessive, >12.0.

#### Microhardness

The surface microhardness was analyzed on circular specimens (n=20) according to specification ISO 4516:2002, using a microdurometer (HMV-2000; Shimadzu, Kyoto, Japan).^15^ Eight random equidistant measurements (40× magnification) were made on each specimen with a Knoop diamond indenter under a load of 25 g for 5 seconds. The microhardness of each specimen was defined by the mean of the eight measurement values obtained.

#### Flexural strength

The flexural strength of rectangular specimens (n=20) was determined by applying the 3-point bending test according to specification ISO 20795-1:2008, using a universal testing machine (DL 2000; EMIC, São Jose dos Pinhais, Paraná, Brazil). With 50 mm of distance between the two supporting points, 50 kg were applied on the center of specimens until they fractured.^21^ Flexural strength was calculated using the peak load applied, span length and specimen widths and thicknesses. The results were expressed in kgf/mm^2^ and converted to MPa. Flexural strength values below 65 MPa were not considered clinically acceptable, in accordance with the ADA Specification No. 12.^14^

#### Impact strength

Rectangular specimens (n=20) were submitted to the Izod type impact test without notch (AIC; EMIC, São José dos Pinhais, Paraná, Brazil), in accordance with specification ASTM D256.^37^ Specimens were placed on the testing machine in a vertical position, so that the 2J-pendulum reached their upper free end. The results were expressed in J/m.

#### Sorption and solubility

Sorption and solubility tests were conducted on disc-shaped specimens (n=20) in accordance with specification ANSI/ADA No.12/1975.^38^ Sorption was determined according to increase in mass per unit volume, while solubility was determined according to loose of mass from specimens. The specimens were weighed (AB204; Metler Toledo, Columbus, Ohio, USA) and placed in desiccators until constant mass was reached (M1). Subsequently, the specimens were immersed in the hygiene solutions and weighed again (M2). After immersions, the specimens were placed in desiccators again, to obtain the constant mass (M3). The sorption and solubility calculations were based on the equations (M2-M1)/V and (M1-M3)/V, respectively; (in which: V=the specimen volume) and expressed in g/cm^3^.

## Data analysis

All statistical comparisons were made by IBM SPSS statistics software (SPSS Statistics for Windows, Version 21.0. Armonk, NY, USA). The datasets of results were evaluated for normality of distribution (Shapiro-Wilk test). Data for *S. mutans* antibiofilm activity, surface roughness, color alteration, microhardness, flexural and impact strength, sorption, and solubility showed a non-normal distribution, thus the Kruskal-Wallis test and Dunn posttest were performed (α=0.05). For *C. albicans* and *C. glabrata* antibiofilm activity and surface roughness under 3D laser confocal microscope, the ANOVA and Tukey post tests (α=0.05) were used. All multiple comparisons were performed with Bonferroni adjustment.

## Results

### Antibiofilm activity

**Figure 1:**
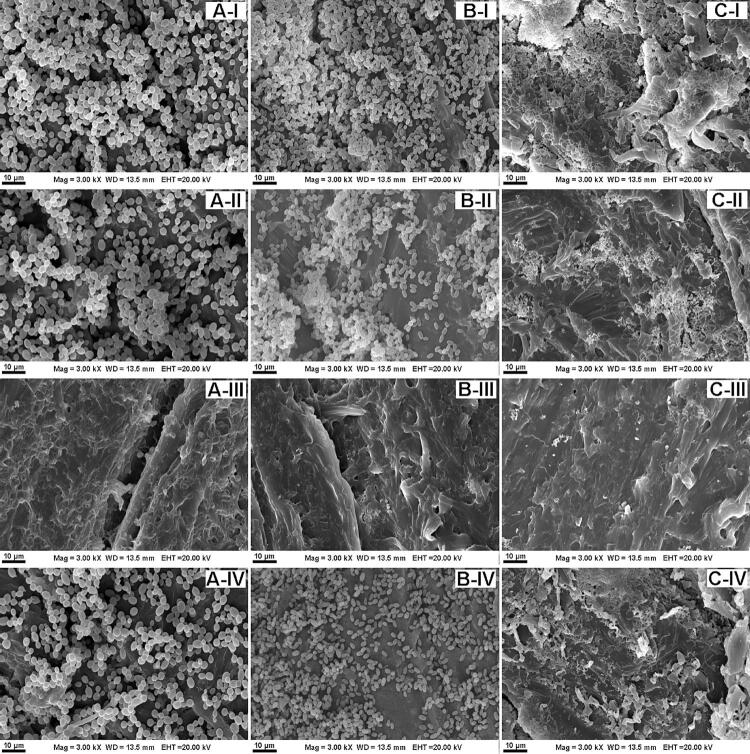
Representative scanning electron microscopy images after immersion in different hygiene solutions. A) *C. albicans*; B) *C. glabrata*; C) *S. mutans*. I) Control; II) RC – *R. communis*; III) SH - Sodium Hypochlorite; IV) EPC – Efferdent Power Clean. Magnification 3000×. Scale bar = 10 µm

Antibiofilm activity was solution dependent. SH reduced the counts of *C. albicans, C. glabrata* and *S. mutans* biofilms on acrylic resin surfaces to zero. Compared with the control group, immersion in EPC exhibited an evident reduction in *S. mutans* biofilm (p=0.001) and immersion in RC promoted favorable antibiofilm activity against *C. glabrata* (p<0.001). Table 1 exhibits Log_10_^CFU^ for the different microorganisms. Figure 1 shows representative scanning electron microscopy images after immersion in the hygiene solutions. The images illustrate a substantial reduction of *C. albicans, C. glabrata* and *S. mutans* biofilm after immersion in SH.

**Table 1:** Log10(CFU+1) of *C. albicans, C. glabrata* and *S. mutans* biofilms after immersion in different hygiene solutions

Microorganisms	Hygiene Solutions	Mean ± SD (Median)	95% IC (Range)	p	Pairwise Comparisions
*C. albicans*	Control	4.93±0.29 (4.89)	4.70; 5.15 (4.54; 5.47)	0.212*	
	RC	4.53±0.57 (4.31)	4.09; 4.97 (3.70; 5.58)		
	SH^Ͱ^	0.00±- (0.00)	-; - (0.00; 0.00)		
	EPC	4.66±0.51 (4.59)	4.27; 5.05 (3.93; 5.45)		
*C. glabrata*	Control	5.98±0.28 (6.04)	5.77; 6.19 (5.45; 6.31)	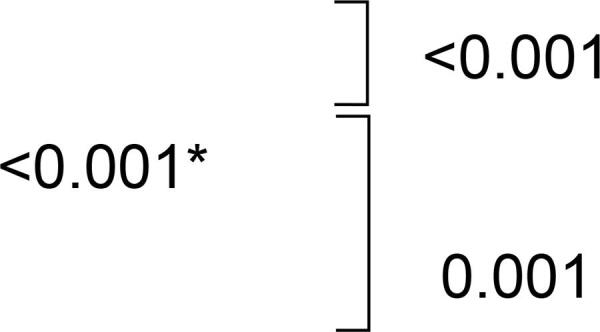	
	RC	5.38±0.32 (5.41)	5.13; 5.63 (4.82; 5.80)		
	SH^Ͱ^	0.00±- (0.00)	-; - (0.00; 0.00)		
	EPC	5.93±0.20 (5.93)	5.78; 6.08 (5.71; 6.26)		
*S. mutans*	Control	6.90±0.37 (7.08)	6.61; 7.18 (6.06; 7.26)	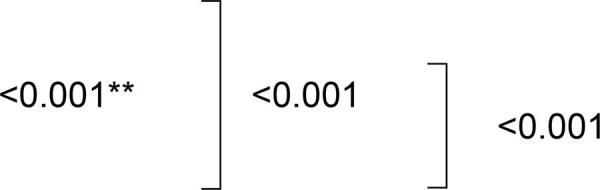	
	RC	6.93±0.34 (7.02)	6.67; 7.19 (6.36; 7.32)		
	SH^Ͱ^	0.00±- (0.00)	-; - (0.00; 0.00)		
	EPC	4.71±0.85 (4.79)	4.06; 5.36 (3.56; 6.01)		

^Ͱ^without CFU values after immersion, *ANOVA and Tukey pos-test; **Kruskal-Wallis test and Dunn pos-test. RC - Ricinus communis; SH - Sodium Hypochorite; EPC - Efferdent Power Clean.

### Surface roughness

**Figure 2:**
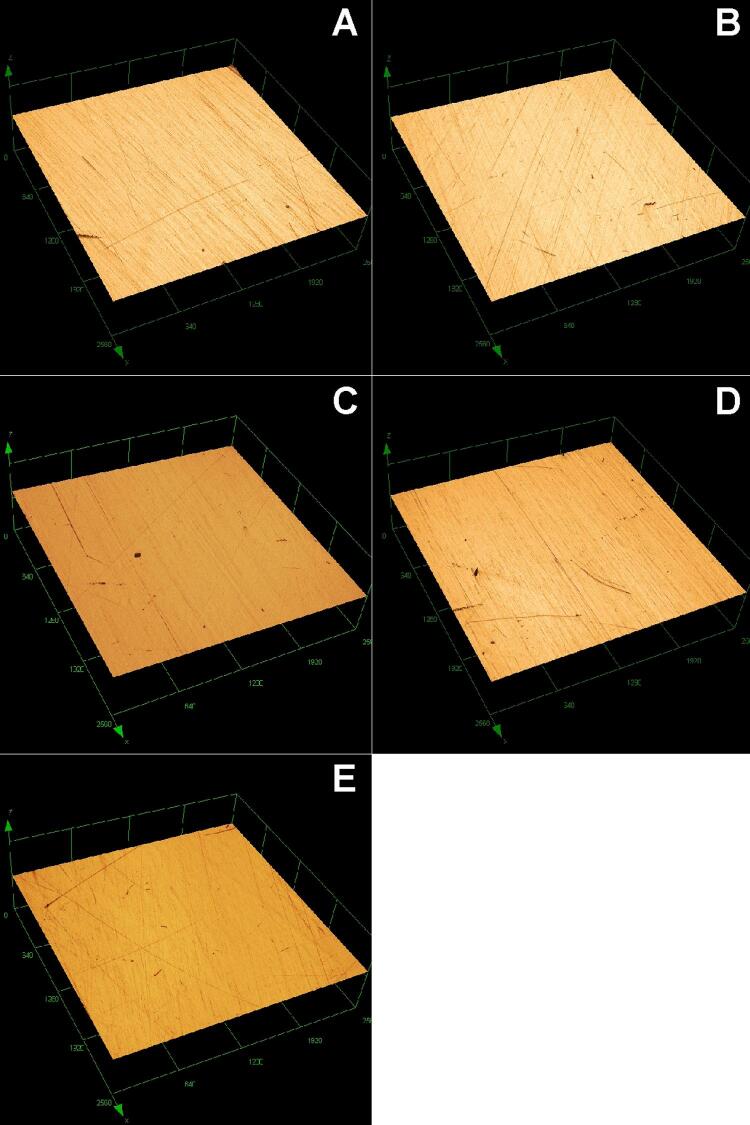
Representative 3D laser confocal microscopy images of specimens after immersion in different hygiene solutions. A) WI - Without Immersion; B) Control; C) RC – *R. communis*; D) SH - Sodium Hypochlorite; E) EPC – Efferdent Power Clean. Magnification 108×

Surface roughness was also solution dependent. After immersion in RC a significant increase in surface roughness was observed (p<0.05). Higher surface roughness values were identified for both evaluation methods, i.e., by rugosimeter (ΔRa) and under 3D laser confocal microscope (Sa). The method of 3D laser confocal microscope seemed to be more sensitive for evaluating the surface roughness. ΔRa (µm) and Sa (µm) values are shown in Table 2. Three-dimensional laser confocal microscopy images are presented in Figure 2. Although a significant alteration could be identified after data analysis (ΔRa and Sa), the roughness surface did not vastly change among the groups (0.00–0.03 µm).

**Table 2:** Effects of different hygiene solutions on physical and mechanical properties of denture base acrylic

Physical and Mechanical Properties	Hygiene Solutions	Mean ± SD (Median)	95% IC (Range)	p	Pairwise Comparisions	
Surface roughness - ΔRa (µm)	Control	0.00±0.03 (0.00)	-0.02; 0.01 (-0.10; 0.02)	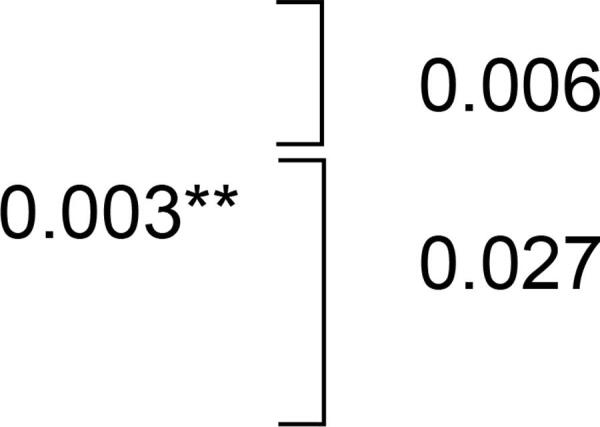		
	RC	0.03±0.04 (0.02)	0.01; 0.06 (-0.01; 0.16)			
	SH	0.02±0.03 (0.01)	0.01; 0.03 (-0.01; 0.08)			
	EPC	0.01±0.02 (0.00)	0.00; 0.02 (-0.02; 0.07)			
Surface roughness - Sa (µm)	WI	0.327±0.065 (0.340)	0.277; 0.376 (0.213; 0.432)	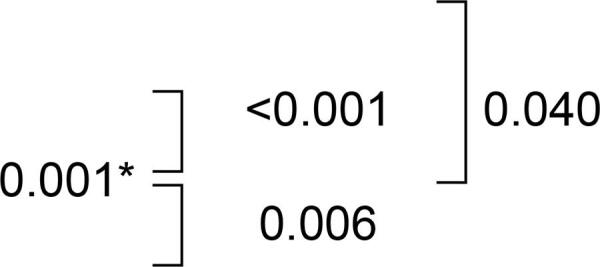		
	Control	0.282±0.045 (0.269)	0.248; 0.317 (0.229; 0.348)			
	RC	0.410±0.074 (0.401)	0.353; 0.467 (0.329; 0.540)			
	SH	0.306±0.032 (0.306)	0.281; 0.331 (0.253; 0.352)			
	EPC	0.339±0.073 (0.351)	0.283; 0.395 (0.262; 0.498)			
Color (ΔE)	Control	0.85±0.66 (0.66)	0.54; 1.16 (0.34; 2.90)	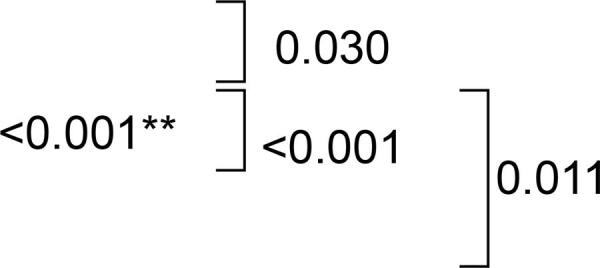		
	RC	1.37±0.84 (1.07)	0.98; 1.76 (0.22; 3.94)			
	SH	0.52±0.21 (0.41)	0.42; 0.61 (0.32; 0.94)			
	EPC	0.97±1.03 (0.61)	0.48; 1.45 (0.19; 4.54)			
Microhardness (ΔHK)	Control	-0.28±1.69 (-0.05)	-1.07; 0.52 (-5.10; 2.10)	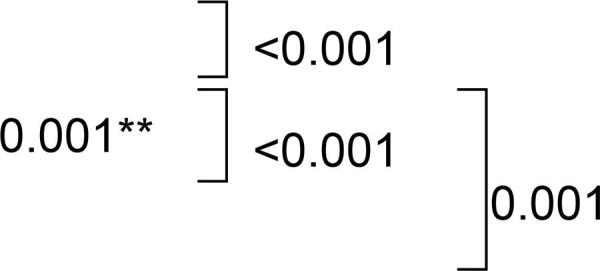		
	RC	2.91±1.82 (2.85)	2.06; 3.76 (-0.10; 6.60)			
	SH	0.16±0.82 (0.05)	-0.23; 0.54 (-1.20; 1.60)			
	EPC	0.54±1.58 (0.25)	-0.20; 1.28 (-1.50; 4.30)			
Flexural Strength (MPa)	WI	96.15±8.53 (98.92)	92.16; 100.14 (78.94; 106.84)	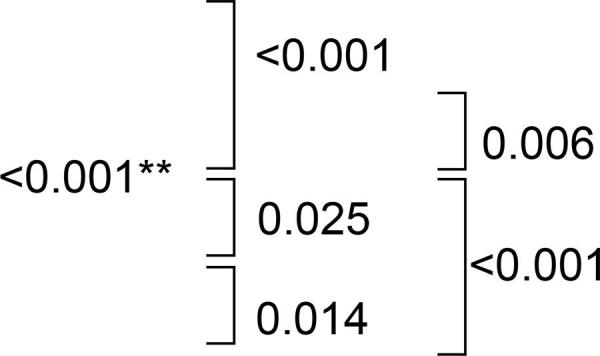		
	Control	89.94±7.58 (90.08)	86.39; 93.49 (72.52; 103.75)			
	RC	74.18±10.25 (74.39)	69.39; 78.98 (56.21; 101.95)			
	SH	88.21±10.16 (86.50)	83.45; 92.97 (67.79; 104.95)			
	EPC	99.73±9.12 (100.94)	95.46; 103.99 (72.17; 114.58)			
Impact Strength (J/m)	WI	165.10±7.59 (164.00)	161.55; 168.65 (156.00; 183.00)	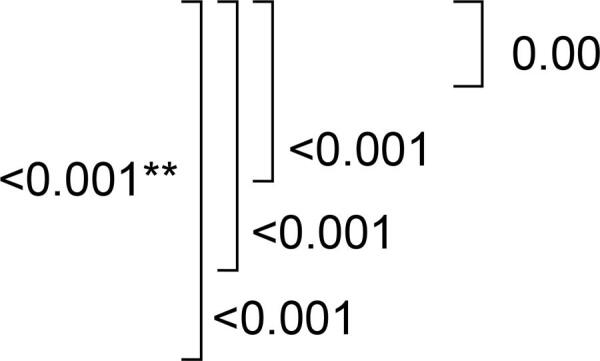		
	Control	146.40±12.56 (147.50)	140.52; 152.28 (128.00; 163.00)			
	RC	132.40 ± 11.68 (135.00)	126.93; 137.87 (110.00; 150.00)			
	SH	141.15±7.10 (144.00)	137.83; 144.47 (128.00; 153.00)			
	EPC	134.10±10.50 (140.00)	129.18; 139.02 (113.00; 144.00)			
Sorption (g/cm^3^)	Control	0.027±0.002 (0.028)	0.026; 0.028 (0.022; 0.031)	0.666**		
	RC	0.026±0.073 (0.028)	-0.008; 0.060 (-0.201; 0.169)			
	SH	0.027±0.003 (0.028)	0.025; 0.029 (0.019; 0.033)			
	EPC	0.028±0.002 (0.028)	0.027; 0.029 (0.024; 0.033)			
Solubility (g/cm^3^)	Control	-0.005±0.016 (-0.001)	-0.012; 0.003 (-0.073; -0.001)	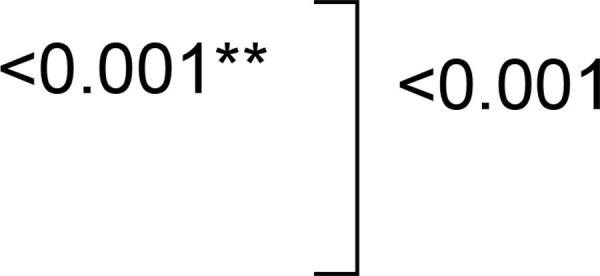		
	RC	-0.002±0.072 (-0.002)	-0.036; 0.032 (-0.147; 0.219)			
	SH	0.002±0.016 (-0.001)	-0.005; 0.010 (-0.002; 0.070)			
	EPC	-0.001±0.000 (-0.001)	-0.001; -0.001 (-0.001; 0.000)			

*ANOVA and Tukey pos-test; **Kruskal-Wallis test and Dunn pos-test. RC - Ricinus communis; SH - Sodium Hypochorite; EPC - Efferdent Power Clean.

### Color Change

According to the CIELAB color scale (ΔΕ), change in color was observed after immersion in all hygiene solutions. Significantly higher ΔE values were observed only for Group RC when compared with Control (p=0.030), SH (p<0.001) and EPC (p=0.011). Nonetheless, when classifying the change in color according to the NBS units the values were less divergent [“trace” (0.0–0.5) for SH (0.43) and “slight” (0.5–1.5) for Control (0.78), EPC (0.89) and RC (1.27)]. Table 2 shows the color change results.

#### Microhardness

The Kruskal-Wallis test showed a significant difference for microhardness (p<0.001). The Dunn test indicated that immersion in RC increased microhardness values, which differed statistically from those of Control (p<0.001), SH (p<0.001) and EPC (p=0.001) (Table 2).

## Flexural strength

Relative to flexural strength, significantly lower values were observed after immersion in RC when compared with control (p=0.006), SH (p=0.025) and without immersion (p<0.001). Whereas higher values were observed after immersion in EPC when compared with RC (p<0.001) and SH (p=0.014). Flexural strength results are illustrated in Table 2.

## Impact strength

A reduction in impact strength was observed after immersion in all hygiene solutions. The group without immersion exhibited the highest impact strength values that differed statistically from those of Control (p=0.003), SH (p<0.001), RC (p<0.001) and EPC (p<0.001) (Table 2).

## Sorption and solubility

For sorption, no changes were observed after immersion in all hygiene solutions (p=0.666) (Table 2). However, for solubility, immersion in RC contributed to greater weight loss than immersion in EPC (p<0.001) (Table 2).

## Discussion

In this study, hygiene solutions were evaluated with regard to antibiofilm activity against *C. albicans, C. glabrata* and *S. mutans* that are microorganisms related to denture biofilm,^1^ and adverse effects on relevant properties of the acrylic resin-based dentures.^15-17,19^ The solutions were applied in short cycles (20 minutes), as a routinely recommended period of immersion, and were not associated with any other hygiene methods, to avoid the synergism of action.^3,23^ The results demonstrated rejection of the null hypothesis, since the solutions showed different effects on antibiofilm activity, surface roughness, color change, microhardness, flexural and impact strength and solubility.

As regards antibiofilm activity, SH was the most effective solution since it reduced the CFU counts of the three microorganisms to zero. Studies have shown the efficacy of SH in removing denture biofilm,^7-10,28^ even in diluted concentrations of 0.1% and 0.2%.^20-22^ Concentrations at 0.5% and 0.25% showed antimicrobial effectiveness against yeast and bacteria (Gram positive and negative), ^4-6,8-11^ and at 0.1% and 0.2%, against *Candida* spp.^20-22^ Moreover, the literature has shown that elevated concentrations, i.e. 1%, should not be used, since these concentrations led to changes in color and flexural strength of the acrylic resins.^13,15^ Concentrations of 0.5% were not deleterious in short cycles (3-20 minutes), or in periods ranging from 180 days^8,12,18^ to 5 years^17^ of daily use. However, this concentration led to changes in color and surface roughness when applied in long cycles (8 hours).^13,16^ A previous study showed that a 0.2% SH solution promoted color change classified as “trace” according to NBS, without changes in the surface roughness and flexural strength of the acrylic resin.^21^ The results of the present study complement these findings, since we observed no changes in microhardness, sorption, solubility and surface morphology. Thre were changes only in impact strength, but within the values established by ISO 1567.^15,37^ Thus, it can be inferred that this solution is effective and can be indicated as a safe denture cleanser in short immersions.

EPC was effective against *S. mutans*, in agreement with a previous study,^29^ but ineffective against *C. albicans* and *C. glabrata*. Peroxide-based solutions, in general, have shown a wide variety of results regarding biofilm removal and antimicrobial action against different microorganisms, with reports of effectiveness,^3,23,30-33^ moderate action^27,29^ and ineffectiveness.^9,25^ These findings may be related to the different methodologies used, such as hygiene protocol used, microorganisms, biofilm recovery rates, biofilm composition^23,26,29^ and even the cleanser itself, since the effectiveness of peroxides is regularly attributed to the active ingredients of the formulations.^24,27,29^ The effectiveness of Efferdent has been associated with the presence of tetrasodium EDTA in its composition.^27^ Its action against *S. mutans*, microorganisms related to the growth of *Candida* in biofilms, was a relevant result and emphasized the importance of using this solution as a denture cleanser. Other protocols of use should be evaluated, since the product has been indicated for longer periods of immersion, with the aim of increasing its effectiveness.^19,31^ With reference to the effects on acrylic resin, similarly to the trend in results observed for SH, there was only a decrease in impact strength. Previous studies have shown that different peroxide-based solutions have decreased the flexural strength of acrylic resins, and promoted color changes classified as “noticeable”, “considerable” and “very”.^14,16,34^ Therefore, these findings may be related to the different compositions of the cleansers. Regarding surface morphology, after immersion in EPC, the acrylic resin acquired a rougher exterior surface. The exact mechanism that alkaline peroxides damage the acrylic resin surface is unclear. It has been proposed that the higher peroxide content and release of oxygen can promote hydrolysis and surface decomposition.^39^

RC showed antibiofilm activity against *C. glabrata*, with a significant decrease in microbial load; however, in agreement with Badaró, et al.^10^ (2017), it was not effective against *C. albicans* and *S. mutans*. Randomized clinical studies have demonstrated moderate efficacy in biofilm removal of RC at 2%, 8% and 10%,^7,10,21,35^ effectiveness of remission of denture stomatitis at 3.3%, 8% and 10%^10,20,36^ and antimicrobial action at 2% and 10%.^6,10^ However, *Candida* spp. have been shown to be more resistant to these solutions, with reports of moderate action of solutions at 2 and 10%^6^ and ineffectiveness of solutions at 3.3% and 8%.^20,36^ These results differed from the findings of this study, since the concentration of 6.25% was effective against *C. glabrata*. Thus, the concentration of RC seems to be a determining factor for effectiveness, so that the ideal amount of water is essential to allow the breakdown of sugar molecules in the cell walls and inactivation of ribosome that promotes cell death.^20,22^ Regarding the adverse effects, in addition to the decrease in impact strength, the solution also led to greater changes in color, microhardness, flexural strength and surface roughness; however, the values were within acceptable clinical limits for each property.^15,17^ Even though the sorption was similar among the groups, RC showed the highest range of values. According to Tuna, et al.^40^ (2008) water and chemicals absorbed from the environment, would cause the decrease of mechanical properties. So, this statement indicates that RC could had bound chemically to acrylic resin, influencing the evaluated properties.

Furthermore, when compared with EPC, the RC solution promoted moderate changes in surface morphology and lower values of mass loss. The few reports in the literature showed that irrespective of the concentration used, RC promoted color changes, however, within the clinical limits established according to the National Bureau of Standards (NBS).^15,18,21^ Changes in surface roughness have also been reported with concentrations at 2% and 10%, as well as decrease in microhardness and flexural strength at 2% concentration.^15,18^ However, when conducted at 8%, no change in the properties of surface roughness and flexural strength was identified.^21^ Therefore, obtaining an ideal concentration of RC is important, not only to guarantee its effectiveness against the denture biofilm, but also to prevent changes in the properties of the acrylic resin-based dentures. According to results obtained, RC can be indicated as a denture cleanser, since it demonstrated antibiofilm action against *C. glabrata*, without showing significant changes in the properties of the acrylic resin.

A limitation of this study were the non-reproducibility of the oral environment. In the oral cavity, the denture is influenced by saliva, acidic foods, masticatory force, and occlusion of the patient and that mixed microbial biofilms were not assessed. In the oral cavity, microorganisms exist in polymicrobial communities and different species interact in a complex manner to modulate biofilm nature. Future studies should involve other concentrations of RC, as well as other peroxide-based formulations, since the results presented in the literature have been inconclusive. An additional factor to be considered is the importance of simulating the 8-hour immersion period, since it is recommended and routinely used by complete denture wearers.^3^

## Conclusion

Based on the experimental conditions of our study, the 0.2% sodium hypochlorite solution was effective against the three tested microorganisms, while Efferdent and 6.25% *R. communis* solutions showed moderate antibiofilm activity against *S. mutans* and *C. glabrata*, respectively. Furthermore, solutions did not significantly alter the acrylic resin properties, after a simulation of five years, as they were considered within acceptable limits.
